# Selective intra-arterial administration of ^18^F-FDG to the rat brain — effects on hemispheric uptake

**DOI:** 10.1007/s00234-014-1335-1

**Published:** 2014-02-14

**Authors:** Fabian Arnberg, Erik Samén, Johan Lundberg, Li Lu, Jonas Grafström, Michael Söderman, Sharon Stone-Elander, Staffan Holmin

**Affiliations:** 1 Department of Clinical Neuroscience, Karolinska Institutet, Stockholm, Sweden; 2 Department of Neuroradiology, Karolinska University Hospital-Solna, SE-17176 Stockholm, Sweden; 3 KERIC, Karolinska University Hospital-Solna, Stockholm, Sweden

**Keywords:** Intra-arterial, Endovascular intervention, 18F-FDG, Radiotracer

## Abstract

**Introduction:**

The purpose of this study was to investigate the radioligand uptake and iodine contrast distribution in the intra- and extracranial circulation of the rat, after intra-arterial injections to the common carotid artery and different parts of the internal carotid artery.

**Methods:**

All animal experiments were carried out in accordance with Karolinska Institutet’s guidelines and were approved by the local laboratory animal ethics committee. We used clinical neurointerventional systems to place microcatheters in the extra- or intracranial carotid artery of 15 Sprague–Dawley rats. Here, injection dynamics of iodine contrast was assessed using digital subtraction angiography. Maintaining the catheter position, the animals were placed in a micro PET and small-animal positron emission tomography (PET) was used to analyze injections [2-^18^F]-2-fluoro-2-deoxy-d-glucose (^18^F-FDG).

**Results:**

Microcatheters had to be placed in the intracranial carotid artery (iICA) for the infusate to distribute to the brain. Selective injection via the iICA resulted in a 9-fold higher uptake of ^18^F-FDG in the injected hemisphere (*p* < 0.005) compared to both intravenous and more proximal carotid artery injections. Furthermore, selective injection gave a dramatically improved contrast between the brain and extracranial tissue.

**Conclusion:**

Intra-arterial injection increases the cerebral uptake of a radiotracer dramatically compared to systemic injection. This technique has potential applications for endovascular treatment of malignancies allowing intra-interventional modifications of injection strategy, based on information on tumor perfusion and risk to surrounding normal parenchyma. Furthermore the technique may increase diagnostic sensitivity and avoid problems due to peripheral pharmacological barriers and first passage metabolism of labile tracers.

## Introduction

Imaging by positron emission tomography (PET) is today an expanding area of experimental and clinical investigations. The development of suitable radiotracers is a crucial factor for this expansion. Currently, radiotracers are primarily designed for intravenous administration. Among the criteria they need to meet are ability to reach and penetrate tissues containing the targets of interest, high affinity for the target, low non-specific binding and limited or measurable metabolism outside the targeted tissue. However, an ability to perform organ-selective administrations could fundamentally alter some of these requirements.

Parallel to the development of PET, endovascular neurointerventional procedures monitored with fluoroscopy and digital subtraction angiography (DSA) have developed to become standard care for a variety of diseases. The technique allows selective navigation to virtually all regions of the vascular system, including the human brain. Implementation of these procedures in nuclear medicine would make it possible to inject radiotracers selectively to targeted regions of interest and thereby bypass many pharmacological hinders to efficient tracer delivery.

Radiotracer-based imaging has occasionally been used together with interventional radiology (IR) to order to, for example, optimize flow rates of carotid drug infusions [1] and monitor regional depositions of chemotherapeutics [2–5]. IR has clinically been combined with single-photon emission computed tomography (SPECT) in brachytherapy of the liver to guide the IA administration and avoid non-target radiation [6, 7]. Inadvertent intra-arterial administration of [2-^18^F]-2-fluoro-2-deoxy-d-glucose (^18^F-FDG) has anecdotally been reported to augment tracer uptake [8, 9]. However, no study has been performed to investigate the possible advantages of intra-arterial administration of ^18^F-FDG on its uptake in targeted tissue. This study was undertaken to investigate how injections of ^18^F-FDG in different locations of the arteries supplying the rat brain would affect tracer uptake and distribution. We hypothesize that the combination of PET, and probably other nuclear medicine techniques, with endovascular interventional systems may increase the value and versatility of this radiotracer as well as other existing or coming radiotracers for clinical use.

## Materials and methods

All animal experiments were carried out in accordance with Karolinska Institutet’s guidelines and were approved by the local laboratory animal ethics committee (N337/10). Fifteen adult male Sprague–Dawley rats (350–400 g) were permitted food and water ad libitum until surgery. Anesthesia was induced using 4 % isoflurane mixed with 96 % O_2_ and subsequently maintained at 2 % isoflurane. Animals were placed on a heating pad and were kept normothermic. Arterial access and endovascular navigation to the carotid artery were performed as described previously, using a 0.40-mm (0.0157 in. microcatheter (Sonic; Balt Extrusion, Montmorency, France) carrying a 0.18-mm (0.007 in. microwire (Hybrid; Balt Extrusion) microcatheter [10].

### Digital subtraction angiography

We placed the microcatheter tip at three different positions, one position in two each of the animals: (1) in the common carotid artery (CCA), (2) in the cervical internal carotid (cICA) although proximal to the branching of the pterygopalatine artery (PPA), (3) distal to the PPA in the intracranial part of the internal carotid artery (iICA). In these different positions, we performed DSA via injections of 100 μl iohexol (Omnipaque; Amersham Health; 180 mg/ml of iodine). The images were analyzed using OsiriX imaging software (OsiriX Foundation).

### Intra-arterial injections of ^18^F-FDG

The ^18^F-FDG used was an aliquot obtained from daily productions for clinical PET at the Karolinska University Hospital and had passed all quality requirements for administrations in humans. Using the same catheterization protocol as described above, the microcatheter tip was positioned in the CCA (*n* = 3 animals) and in the iICA (*n* = 3 animals). ^18^F-FDG (10–20 MBq, 500 μl) followed by a saline flush of 300 μl was injected in each animal at a rate of 0.125 ml/min. In three additional animals, ^18^F-FDG was injected via the tail vein.

### Imaging and data analysis

In vivo PET investigations were performed on a MicroPET Focus 120 (CTI Concorde Microsystems). PET data were processed with MicroPET manager and evaluated using the Inveon Research Workplace (Siemens Medical Solutions) software. Data were collected continuously from the time of injection for 25 min and were corrected for random, dead time and decay. Images were reconstructed by standard 2D filtered back projection using a ramp filter. Regions of interest were drawn manually to cover cortical areas supplied by the ICA.

### Statistical methods

The SPSS statistical packet (SPSS Mac OS X version 21, SPSS Inc., Chicago, IL, USA) was used for the calculations. Radioactivity levels in cerebral hemispheres at 25 min after injection were compared using analysis of variances (ANOVA), followed by Tukey's post hoc test. Any *p* value <0.05 was considered significant.

## Results

### Selective intra-arterial injection of ^18^F-FDG

**Fig. 1:**
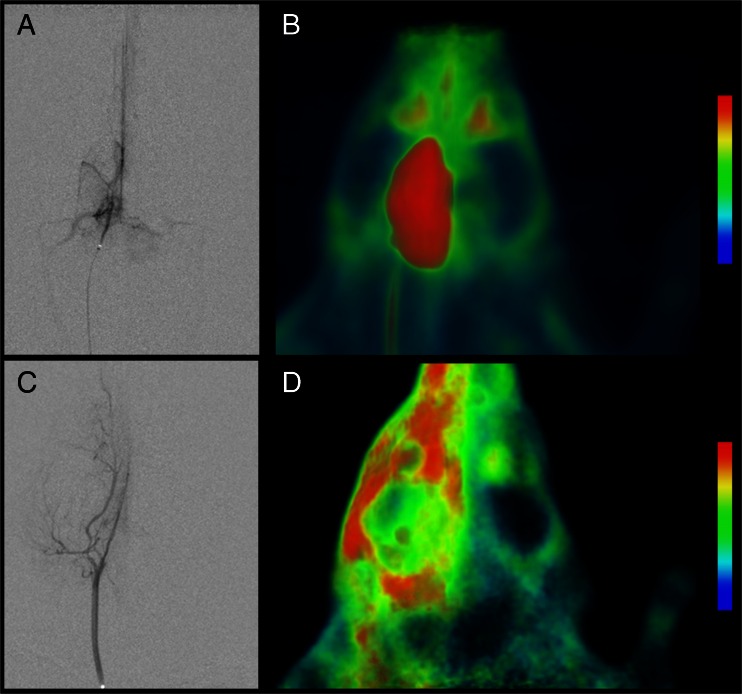
Distribution of ^18^F-FDG and iodine contrast media following selective intra-arterial injections in the internal and common carotid arteries. **a** Digital subtraction angiography (*DSA*) and **b** ^18^F-FDG PET, following injection in the left internal carotid artery (*iICA*), showing perfusion and uptake in the left hemisphere. **c** DSA and **d** ^18^F-FDG PET, following injection in the left common carotid artery, showing perfusion of extra- and intracranial vasculature and ^18^F-FDG distributions, respectively. PET images are volume-rendering technique 3D projections and are summed over 25 min

**Fig. 2:**
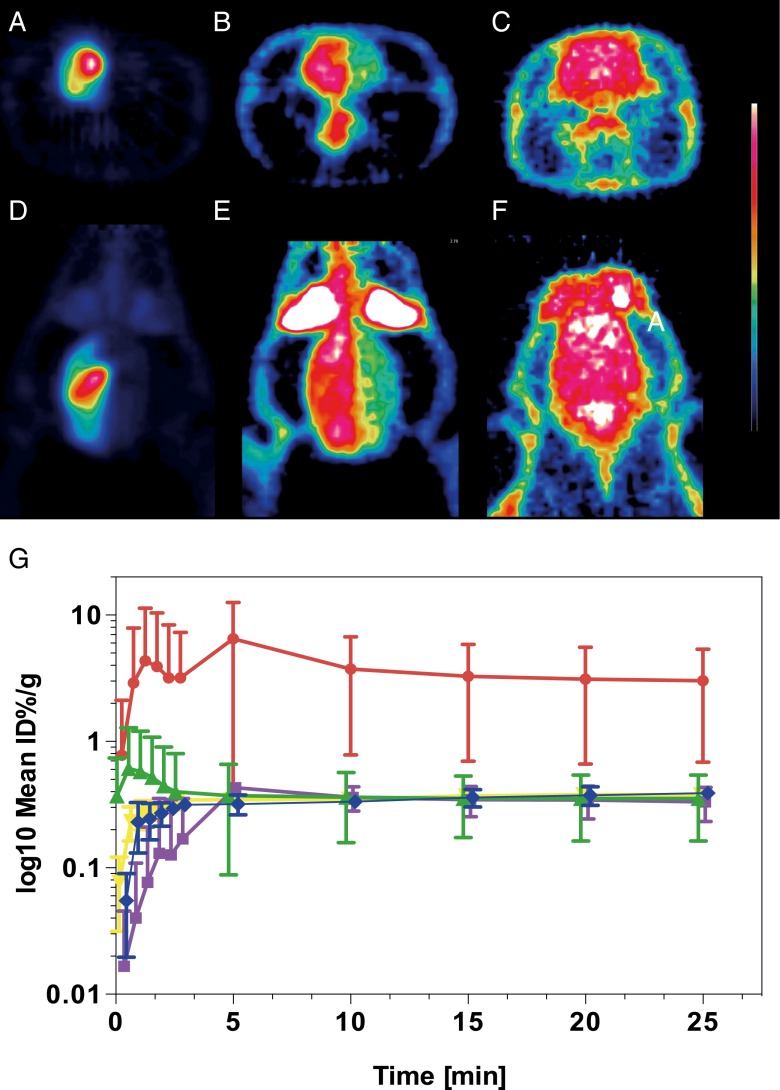
Distribution and changes in tissue radioactivity of ^18^F-FDG following selective intra-arterial injections in the internal and common carotid arteries and systemic injections in the tail vein. ^18^F-FDG PET, transaxial and coronal planes, following: **a**, **d** injection in the left internal carotid artery (*iICA*); **b**, **e** injection in the common carotid (CCA); **c**, **f** injection in the tail vein. **g** Changes in tissue radioactivity over time expressed as percentage of the injected dose per gram tissue (%ID/g; *Y* axis = log_10_ mean %ID/g) following: injection in the iICA in the left (ICA LH, *red dot*) and the right (ICA RH, *purple square*) cerebral hemispheres; following injection in the left CCA in the left (CCA LH, *green triangle*) and the right (CCA RH, *yellow inverted triangle*) cerebral hemispheres; following i.v. injection (IV, *blue rhombus*). The differences in mean values at 25 min are significant between ICA LH and ICA RH (*p* = 0.009), ICA LH and CCA LH (*p* = 0.008) and between ICA LH and IV (*p* = 0.004). PET images are summed over 25 min. Error bars indicate ±1 SD

Injections of ^18^F-FDG were performed with the tip of the microcatheter located in the CCA (*n* = 3), in the iICA (*n* = 3) or in the tail vein (*n* = 3). Catheter tip placement in the CCA resulted in a markedly more intense uptake of ^18^F-FDG in the ipsilateral extra- and intracranial tissues compared to contralateral tissues (Figs. 1d and 2b, e). With this catheter position, the uptake was most prominent in the extracranial compartment (Fig. 1d); the anatomical distribution of the tracer was identical to the opacification following injections of iodine contrast in the CCA, showing that intra-arterial injection in this location resulted in distribution predominantly in the extracranial space (Fig. 1c, d). However, even with this catheter position, the uptake in the hemisphere ipsilateral to the injection tended to be higher than the uptake in the contralateral hemisphere, although without reaching significant difference at equilibrium (Fig. 2b, e). On the other hand, placement of the catheter tip in the iICA resulted in an intense uptake in the left cerebral hemisphere only, corresponding to 9.2 times higher uptake compared to the CCA injection at equilibrium after 25 min (*p* = 0.009) (Figs. 1b and 2a, d, g). Also, when comparing the ipsilateral cerebral hemisphere to the contralateral hemisphere after iICA injection, the difference was 9.2 times higher (*p* = 0.008) (Fig. 2a, d, g). The areas with high uptake corresponded to the cortical areas supplied by the iICA (Fig. 2a, d). The cerebral uptake of ^18^F-FDG after iv injection was 8.69 times lower than after the iICA injection (*p* = 0.004) (Fig. 2a, c, d, f, g). Extra-cranial ^18^F-FDG uptake was lower after iICA and CCA injections than after the iv injection (Fig. 2a–f). Systemic uptake levels were higher after the CCA injection than after the iICA injection, consistent with a higher extraction of ^18^F-FDG in the brain compared to soft tissue (Fig. 2a, b, d, e).

### Injections of iodine contrast media to the carotid artery

DSA of the CCA and its branches confirmed the already well defined anatomy of the external carotid artery (ECA), the cICA, the iICA and the PPA of the rat [11] (Fig. 2a, d). The approximate relative diameters of the CCA:ECA:cICA:iICA:PPA were in concordance with anatomic studies [11], i.e., 1.0:0.8:0.7:0.5:0.5, respectively. Injections of iodine contrast media in the CCA resulted in reproducible opacification of the ECA and PPA territories whereas opacification of the iICA was intermittent and weaker than the ECA and PPA (Fig. 1c). Similarly, iodine contrast media injections in the cICA resulted in reproducible opacification of the PPA whereas opacification of the iICA territory was intermittent and weaker than the PPA. Injections directly in the iICA branch, however, resulted in robust and reproducible opacification of cortical territories, clearly showing the anatomy of the intracerebral arterial branches (Fig. 1a). Thus, for administering contrast media (and other substances) selectively to the brain itself, it was necessary to position the catheter tip in the iICA (distal to the PPA).

## Discussion

In this paper, we suggest a combination of PET and the endovascular neurointerventional suite to provide intra-procedural nuclear medicine imaging during or after selective intra-arterial injection of a radioligand. We used the most commonly used clinical radiotracer ^18^F-FDG with a high utilization in the brain, so the consequences of intra-arterial administration at different positions in the carotid vessel could be readily visualized. The main determinants for a sensitive and specific PET visualization of CNS pathology are the radiotracer properties, the local concentrations and the proliferative, metabolic and receptor state of the target tissue. The radiotracer needs to provide a clear contrast between normal and pathological tissues even in situations when the pathologic tissue has poor blood perfusion, altered membrane diffusion, blood–brain barrier (BBB) breakdown or aberrant metabolism. At the same time, the administered amounts of radioactivity must be kept at as low as reasonably possible, especially when using therapeutic radionuclides, whose off-target tissue expositions can be dose limiting. We show that selective intra-arterial injection of ^18^F-FDG resulted in a factor of 9 increase in tracer uptake in the targeted tissue compared to intravenous injection and also compared to less selective intra-arterial administrations.

We started with analyzing the dynamics of injecting iodine contrast in different positions of the carotid artery. In the rat, the CCA directs blood flow to the extracranial circulation to a larger extent than in man [11]. Furthermore, internal carotid injection dynamics involve streaming of the infusate that results in heterogeneous delivery to the regions supplied by the carotid artery [12]. We show that injections at a slow rate in the CCA or the proximal part of the internal carotid artery, before the origin of the pterygopalatine artery, result in streaming of the major part of the infusate to the extracranial circulation. This finding emphasizes the need to consider un-voluntary heterogeneous delivery to the rat brain as a result of streaming phenomena when injecting radiotracers or other substances in more proximal parts of the carotid artery. On the other hand, selective administration via the microcatheter placed in the iICA gave a robust exposure of radiotracer and other agents to the vascular bed of the brain.

Increased local uptake of ^18^F-FDG following unintentional intra-arterial injections to the forearm in patients was documented by Kumar in 2009 [8] and reviewed by Zhu et al. in 2011 [9]. To our knowledge, the differences in ^18^F-FDG uptake following intra-arterial injections to the brain compared to intravenous injections have not been described previously. In this paper we demonstrate the intra- and extracranial tissue distribution of ^18^F-FDG following intra-arterial injections in different parts of the carotid arterial tree. We show that it is possible to enhance the uptake of ^18^F-FDG by a factor of nine in the brain by intra-arterial injection and dramatically improve the contrast between brain and extracranial tissues. The findings of this paper are intriguing, given the advancements of endovascular interventional neuroradiology in the treatment of acute stroke in recent years [13] and the potential for using ^18^F-FDG as a diagnostic tool in brain ischemia [14]. Previously, PET has been used to evaluate the pharmacokinetic advantages of intra-arterial chemotherapy to malignant brain tumors [2, 3]. Currently, superselective cerebral infusions of monoclonal antibody therapies are evaluated in the treatment of malignant gliomas (MG) [15]. Preoperative determination of perfused areas in MG by SPECT has been shown to be useful [16]. Intra-arterial chemotherapy has recently emerged as a safe and feasible procedure for eye-conserving management of retinoblastoma [17–19]. However, superselective catheterization of and drug delivery in end-arteries to the CNS require rigorous training and preparation. This model may serve as a simulation of microcatheter handling and evaluation of different injection techniques. Also for neuroscience and psychiatric research, and maybe treatment, the possibility to selectively administer a ligand to different sub-lobar compartments of the brain could be an important tool. In general, the principle of superselectively administering a wide range of radioligands could markedly increase the sensitivity and resolution of nuclear medicine studies in diverse diagnostic and therapeutic approaches. One major obstacle in molecular MRI is the weakness of conventional contrast agents in relation to some molecular epitopes. Interventional techniques may be a useful tool to overcome this problem by selective administration of targeted nanoparticles under MRI [20, 21]. For studies requiring radiotracers that undergo an important first passage metabolism in, for example, the liver, this principle could be of utmost importance, as we have recently demonstrated using these techniques [22].

## Conclusion

We investigated radioligand uptake in the brain after intra-arterial injections to the CCA and different parts of the internal carotid artery. We show that the intra-arterial injection of ^18^F-FDG results in markedly different hemispheric uptakes in the brain compared to intravenous delivery and that the exact intra-arterial catheter tip location in the vascular system has important implications for the parenchymal uptake of ^18^F-FDG. We believe that the combination of nuclear medicine and endovascular interventional techniques could be of use in superselective cytostatic delivery to brain tumors, where intra-interventional radioligand administration would give information on injection selectivity of tumor tissue versus normal parenchyma exposure. Intra-arterial techniques may also make the development of new radiopharmacons possible, leading to new applications in imaging neurological and other diseases. It may be possible to decrease the total dose of a given radiotracer while dramatically increasing the local concentration. This is of importance when using therapeutic radionuclides, whose off-target tissue expositions can be dose-limiting.
